# Aminoglycoside Resistance and Possible Mechanisms in *Campylobacter* Spp. Isolated From Chicken and Swine in Jiangsu, China

**DOI:** 10.3389/fmicb.2021.716185

**Published:** 2021-10-08

**Authors:** Xiaoyan Zhang, Qian Zhou, Mengjun Tang, Junhua Pu, Jing Zhang, Junxian Lu, Yunzeng Zhang, Yushi Gao

**Affiliations:** ^1^Supervision, Inspection & Testing Centre for Poultry Quality (Yangzhou), Ministry of Agriculture, Jiangsu Institute of Poultry Science, Yangzhou, China; ^2^Jiangsu Key Lab of Zoonosis, Jiangsu Co-Innovation Center for Prevention and Control of Important Animal Infectious Diseases and Zoonoses, Yangzhou University, Yangzhou, China

**Keywords:** *Campylobacter*, aminoglycoside resistance, natural transformation, MLST, Food Safety

## Abstract

*Campylobacter* is a major food-borne pathogen in humans, and previous studies reported a high prevalence of gentamicin-resistant *Campylobacter* isolates from food-producing animals in China. This study aimed to investigate the aminoglycoside resistance of *Campylobacter* isolated from chicken and swine in Jiangsu province, China and understand the possible mechanisms responsible for aminoglycoside resistance. One hundred and eighty-five *Campylobacter* isolates of chicken and swine origins in 2017 and 2018 were analyzed for gentamicin and kanamycin resistance. Some aminoglycoside resistance genes were selected for PCR detection in all strains. The genomic DNAs of two strains with high resistance to gentamicin were used as donors to subject *C. jejuni* NCTC11168 to natural transformation. The transformants were investigated by whole-genome sequencing and analyzed comparatively with *C. jejuni* NCTC11168. In total, 30.5% (29/95) of *C. jejuni* isolates and 42.2% (38/90) of *C. coli* isolates were resistant to gentamicin and kanamycin. The prevalence of the *aph(2")-If* gene and *aac(6')-Ie/aph(2")-Ia* gene was 65.4% (121/185) and 36.2% (67/185) in *Campylobacter* isolates, respectively. The *aadE-sat4-aphA-3* cluster was identified in 8.7% (8/92) and 20.4% (19/93) of all *Campylobacter* isolates in each year. With each donor DNA, aminoglycoside-resistant transformants were obtained. The transformants showed ≥128-fold increases in the MICs of gentamicin, kanamycin, and tobramycin. A 5200-bp segment was found to be inserted between the highly conserved genes *Cj0299* and *panB* of *Campylobacter*. A total of 9.7% (18/185) strains showing high resistance to aminoglycosides had this segment by PCR detection. The genetic diversity of the insertion-fragment positive strains was determined by MLST, and seven sequence types were identified for these strains.

## Introduction

*Campylobacter jejuni* and *Campylobacter coli* are the main pathogens that cause sporadic gastroenteritis worldwide (Costa and Iraola, 2019; Man, 2011). In 2010, *Campylobacter* was estimated by laboratory confirmation to cause the highest number of food-borne bacterial infections globally (Noordhout et al., 2017). With a laboratory-modified isolation kit based on a membrane filter method (ZC-CAMPY-002, Qingdao Sinova Biotechnology Co., Ltd., Qingdao, China) has been extensively used to isolate *Campylobacter* from diarrheic patients in Chines CDC’s surveillance project, the *Campylobacter* isolation in sporadic diarrheal cases significantly increased (Li et al., 2018). But compared with other countries, the outbreak caused by *Campylobacter* in China is rare (Li et al., 2020). Most *Campylobacter* enteritis cases are usually mild and self-limiting and do not require antimicrobial therapy; however, for severe or prolonged cases, antibiotic treatment is needed. Fluoroquinolones and macrolides are commonly used to treat campylobacteriosis, but aminoglycosides are used in systemic infections, such as bacteremia (Payot et al., 2006; Blaser and Engberg, 2008). Aminoglycosides are important veterinary antimicrobials in all major food-producing animals to treat infections and are classified by the WHO as important antimicrobials for human medicine (Giguère et al., 2013; World Health Organization, 2019). The extensive use of antibiotics in food-animal production has led to an increase in antimicrobial-resistant strains of *Campylobacter* (Alfredson and Korolik, 2007).

Among the known mechanisms of acquired aminoglycoside resistance, the enzymatic modification is the most common mechanism for the inactivation of aminoglycosides in many bacterial species, including *Campylobacter* spp. (Vakulenko and Mobashery, 2003; Ramirez and Tolmasky, 2010). The aminoglycoside-modifying enzymes are divided into three main classes based on the reactions they catalyze as: aminoglycoside acetyltransferases (AAC), aminoglycoside nucleotidyltransferases, and aminoglycoside phosphotransferases (APH) (Ramirez and Tolmasky, 2010). Numerous aminoglycoside resistance genes have been reported on mobile genetic elements and transposons (Lee et al., 2002; Nirdnoy et al., 2005). Gentamicin resistance genes including *aacA4*, the bifunctional gene *aac(6')-Ie/aph(2")-Ia* (also named *aacA-aphD*), *aph(2")-If*, and *aph(2")-Ig* have been detected in *Campylobacter* (Chen et al., 2013; Zhao et al., 2015). A unique genetic structure containing the aminoglycoside resistance gene cluster *aadE-sat4-aphA-3* and *aacA-aphD* has been identified on the chromosome of *C. coli* (Qin et al., 2012). Recently, a high prevalence and predominance of the *aph(2")-If* gene has been reported in *Campylobacter* (Yao et al., 2017). The *aph(2")-If* shows resistance to gentamicin and other aminoglycosides, such as kanamycin, sisomicin, and tobramycin (Toth er al., 2013). A novel streptomycin resistance gene has also been described, and its widespread presence among *C. coli* may partly account for the streptomycin resistance (Olkkola et al., 2016).

Gentamicin resistance in *Campylobacter* isolated from various livestock commodities along the food-producing continuum (“farm to fork”) and in humans is rare and stable in most countries. In Netherlands in 2017, no gentamicin resistance was found in *Campylobacter* isolates from cattle, pigs, and poultry. The resistance to streptomycin varies between 0 and 2.6% in *C. jejuni* isolates from broilers (European Food Safety Authority (EFSA), 2017). The level of resistance to streptomycin and gentamicin is low (0–1%) for *C. jejuni* from broilers and cattle in Denmark (DANMAP, 2019). Based on a report of the National Antimicrobial Resistance Monitoring System, gentamicin resistance in *Campylobacter* in the United States was rare before 2007 but has increased rapidly and been detected in 12.2% and 18.1% of human isolates and retail isolates, respectively (National Antimicrobial Resistance Monitoring System: Enteric Bacteria, 2011; National Antimicrobial Resistance Monitoring System: Retail Meat Annual Report, 2011). In China, several aminoglycoside agents have been used in conventional broiler and swine productions, and the prevalence of gentamicin-resistant *Campylobacter* from broiler and swine is much higher than that in the United States. A recent study has shown that over 60% of *Campylobacter* isolated from swine and chicken are resistant to gentamicin (Yao et al., 2017). Up to 95% of *C. coli* isolated from chicken and 23% from swine are gentamicin resistant (Chen et al., 2010; Qin et al., 2011; Ma et al., 2014).

In the present study, the prevalence of gentamicin and kanamycin resistance and the associated aminoglycoside resistance genes were analyzed in *Campylobacter* isolated from chicken and swine in Jiangsu province, China. We found a gene segment which could be transferred from *C. jejuni* or *C. coli* to a *C. jejuni* strain by natural transformation, resulting in a drastic increase in aminoglycoside resistance. These findings suggested that the responsible use of aminoglycosides is highly important in safeguarding public health in China.

## Materials and Methods

### *Campylobacter* Strains and Aminoglycoside-Susceptibility Testing

A total of 185 *Campylobacter* isolates (95*C. jejuni* and 90*C. coli* isolates) was investigated in this study. All *Campylobacter* isolates were recovered in 2017 and 2018 from cloacal swabs of chickens and feces of swine from Jiangsu province, eastern China, during our laboratory annual antimicrobial resistance surveillance program. Strains in 2017 were isolated from chicken cloacal swabs and chicken meat: 67 *Campylobacter* strains from chicken cloacal swabs (samples number=150) and 25 *Campylobacter* strains from chicken meat (samples number=80). The strains in 2018 were isolated from chicken cloacal swabs and pig animal feces: 73 *Campylobacter* strains from chicken cloacal swabs (samples number=150) and 22 *Campylobacter* strains from pig fresh feces (samples number=100). Chicken cloacal swabs were collected from 15 chicken farms in each year. From each farm, 10 cloacal swabs were taken from randomly selected animals. Chicken meat samples were collected from 16 supermarkets located in seven districts in Jiangsu Province. From each supermarket, five chicken meat samples were selected randomly. The pig fresh feces were collected from randomly selected animals in 10 pig farms located in Jiangsu province. All isolates were frozen at −80°C in a brain heart infusion broth with 20% glycerol. *Campylobacter* were grown on Mueller Hinton (MH) agar (Difco, MD, United States) supplemented with 5% sheep blood under microaerophilic conditions (85% nitrogen, 10% carbon dioxide, and 5% oxygen) at 42°C. The MICs of gentamicin and kanamycin for *Campylobacter* were determined by the standard agar dilution method according to the guidelines of the (Clinical and Laboratory Standards Institute, 2016). The isolates which showed resistance to gentamicin and kanamycin were further examined for their susceptibility to amikacin, tobramycin, and streptomycin. The reference strain *C. jejuni* ATCC 33560 was used as a quality-control strain. The above experiments were repeated twice to confirm the reproducibility of the MIC data. The CLSI MIC interpretive criteria for resistance of antimicrobial agents were used. Antimicrobial agents were obtained from the Biomed Biotechnology Company.

### Identification of Aminoglycoside Resistance Genes in *Campylobacter* Isolates

The known aminoglycoside resistance genes *aph(2")-If*, *aacA4*, and *aac(6')-Ie/aph(2")-Ia*, as well as the *aadE-sat4-aphA-3* gene cluster, were selected for PCR detection in all strains. Genomic DNA was isolated from the strains by using a TIANamp Bacteria DNA purification kit (TIANGEN, Beijing, China). Amplifications were carried out in a 25μl PCR mixture composing 12.5μl of Ex-Tag (Takara, Dalian, China), 1μl of each primer, 1μl of chromosomal DNA template, and 9.5μl of sterile distilled water. The amplifications were carried out on a thermal cycler using the following parameters: 94°C for 5min, followed by 30cycles of 94°C for 40s, annealing temperature specific to the primer pair for 30s and 72°C for 1.5min, and final extension at 72°C for 10min. The primers of these genes and the annealing temperatures for the different target genes are listed in Table 1. The amplified products were separated by gel electrophoresis on 1.0% agarose gels, stained with GelRed, and visualized under UV light.

**Table 1:** PCR primers used in this study.

Primers	Sequence (5'-3')	Annealing temperature (°C)	Product size (bp)	References
*aph(2")-If*-F	AAGGAACTTTTTTAACACCAG	50	420	Zhao et al., 2015
*aph(2")-If*-R	CCWATTTCTTCTTCACTATCTTC			
*aacA*4-F	ATCTCATATCGTCGAGTGGAC	50	440	Zhao et al., 2015
*aacA*4-R	CGTGTTTGAACCATGTAC			
*aac(6')-Ie/aph(2")-Ia*-F	ACAGAGCCTTGGGAAGATGAAG	54	1,106	Zhao et al., 2015
*aac(6')-Ie/aph(2")-Ia*-R	TGTTCCTATTTCTTTCTTCACTATC			
*aadE-sat4-aphA-3*-F	CGAGGATTTGTGGAAGAGGCTT	55	1,000	Qin et al., 2012
*aadE-sat4-aphA-3*-R	TTCCTTCCAGCCATAGCATCATG			
*cj0299*-F	GTGCCGCTTGTATTATACTC	55	unknown	Yao et al., 2017
*panB*-R	GGCATATCAGCAAGTACGAAAGAC			

### Natural Transformation and Whole-Genome Sequencing

A natural transformation assay was performed according to the method described by Wang and Taylor with minor modifications (Wang and Taylor, 1990). The genomic DNAs of aminoglycoside-resistant *Campylobacter* isolates served as the donors, whereas the aminoglycoside-sensitive strain of *C. jejuni* NCTC11168 served as the recipient. In a typical procedure, the fresh recipient cells were spread on MH agar at about 1×10^8^ cells per plate and cultured for 6–8h (at 42°C under microaerobic conditions). Then, 1μg of genomic DNA of the donor strain was added to the inoculated agar followed by 5h of continuous incubation at 42°C under microaerobic conditions. The cells were harvested and plated on the selective MH agar plate containing kanamycin (60μg/ml), and the plate was further cultured for 48–72h at 42°C under microaerobic conditions. The recipient without donor DNA cultured on the same MH agar served as a negative control. Single colonies of transformants were selected and sub-cultured on gentamicin-containing plates for purity. The MICs of the aminoglycoside (gentamicin, kanamycin, amikacin, tobramycin, and streptomycin) resistance for the transformants were determined by the standard agar dilution method according to the guidelines of the CLSI (Clinical and Laboratory Standards Institute, 2016). Subsequently, two transformants were selected for whole-genome sequencing on an Illumina HiSeq 2,500 platform (Novogene, Beijing, China). The generated 150bp pair-end reads were trimmed and quality controlled, and the clean reads were assembled using SPAdes software. The draft genomes of the transformants were compared with NCTC11168 using Mauve.

### PCR Detection of the Insert Fragment

According to the insertion site of the fragment, the primers were synthesized following a previous method (Yao et al., 2017) (Table 1). The unknown insertion fragment was detected in *Campylobacter* isolates by PCR. The EmeraldAmp PCR Master Mix (RR300A) (TaKaRa, Dalian, China) was composed of 12.5μl of Hot start DNA polymerase, 1μl of each primer, 1μl of chromosomal DNA template prepared as previously described, and 9.5μl of sterile distilled water. PCR was carried out according to the instructions of RR300A. The PCR products were separated as described above.

### MLST and Aminoglycoside-Susceptibility Analysis of Insertion-Fragment Positive Isolates

MLST analysis of insertion-fragment positive *Campylobacter* strains was performed following a previously described method (Dingle et al., 2005). DNA was extracted from the selected strains by using a commercial DNA Kit (Tiangen Biotech Inc., Beijing, China). Primer sequences were obtained from http://pubmlst.org/*Campylobacter*. The nucleotide sequences of the amplicons were determined by Sangon Biotech (Shanghai, China). Allele numbers and sequence types (STs) were assigned using the *Campylobacter* PubMLST database. Subsequently, the resistance to multiple aminoglycoside antimicrobials of the insertion-fragment positive strains was investigated.

## Results

### Aminoglycoside Resistance in *Campylobacter* Isolates

**Figure 1:**
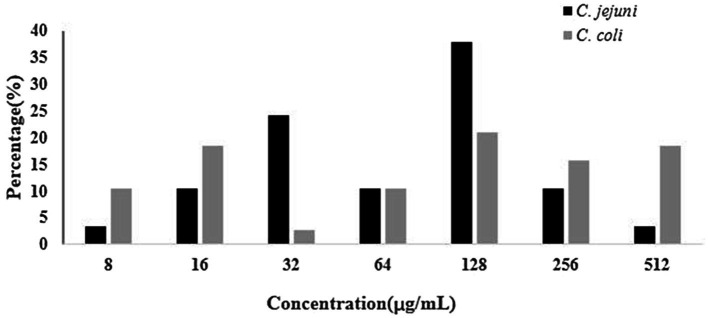
Distributions of gentamicin MICs of gentamicin-resistant *Campylobacter*.

A total of 36.2% (67/185) *Campylobacter* isolates was resistant to both gentamicin and kanamycin. Among the 92 *Campylobacter* isolates tested in 2017 (*C. jejuni*, 51; *C. coli*, 41), 28 (30.4%, including 13*C. jejuni* and 15*C. coli*) were resistant to the above two antibiotics. However, in 2018, among 93 *Campylobacter* isolates tested (*C. jejuni*, 44; *C. coli*, 49), 39 (41.9%, including 16*C. jejuni* and 23*C. coli*) showed resistance to these two drugs. The proportions of gentamicin and kanamycin-resistant *Campylobacter* strains ranged from 28.1% to 60.0% among the seven regions. The rate of resistance of *Campylobacter* to gentamicin and kanamycin in 2018 was higher than that in 2017. A higher percentage of *C. coli* than *C. jejuni* isolates was resistant to gentamicin and kanamycin (Table 2). The distributions of gentamicin MICs of gentamicin-resistant *Campylobacter* are shown in Figure 1, which revealed that the strains with an MIC value of 128μg/ml accounted for a large proportion.

**Table 2:** Prevalence of gentamicin and kanamycin resistance and the associated resistance genes.

Year	Location of isolates	Host	No. of gentamicin and kanamycin-resistant isolates/total no. of isolates (%)		No. of *aadE-sat4-aphA-3*-positive isolates/total no. of isolates (%)		No. of *aph(2")-If*-positive isolates/total no. of isolates (%)		No. of *aac(6')-Ie/aph(2")-Ia*-positive isolates/total no. of isolates (%)	
			*C. jejuni*	*C. coli*	*C.jejuni*	*C. coli*	*C. jejuni*	*C. coli*	*C. jejuni*	*C. coli*
2017	YZ	chicken	3/10(30.0)	4/18(22.2)	0/10(0)	1/18(5.6)	5/10(50.0)	14/18(77.8)	2/10(20.0)	14/18(77.8)
	HA	chicken	3/12(25.0)	4/12(33.3)	1/12(8.3)	4/12(33.3)	5/12(41.7)	8/12(66.7)	0/12(0)	4/12(33.3)
	SQ	chicken	3/10(30.0)	2/4(50.0)	0/10(0)	2/4(50.0)	6/10(60.0)	4/4(100.0)	1/10(10.0)	4/4(100.0)
	YC	chicken	1/6(16.7)	3/3(100.0)	0/6(0)	0/3(0)	1/6(16.7)	3/3(100.0)	0/6(0)	2/3(66.7)
	XZ	chicken	2/6(33.3)	1/3(33.3)	0/6(0)	0/3(0)	3/6(50.0)	2/3(66.7)	0/6(0)	1/3(33.3)
	CZ	chicken	1/5(20.0)	1/1(100.0)	0/5(0)	0/1(0)	1/5(20.0)	1/1(100.0)	0/5(0)	1/1(100.0)
	NT	chicken	0/2(0)	0/0(0)	0/2(0)	0/0(0)	0/2(0)	0/0(0)	0/2(0)	0/0(0)
Total			13/51(25.5)	15/41(36.6)	1/51(2.0)	7/41(17.1)	21/51(41.2)	32/41(78.1)	3/51(5.9)	26/41(63.4)
			28/92 (30.4)		8/92 (8.7)		52/92 (57.6)		29/92 (31.5)	
2018	YZ	chicken	4/19(21.1)	5/10(50.0)	0/19(0)	0/10(0)	17/19(89.5)	10/10(100.0)	12/19(63.2)	6/10(60.0)
	HA	chicken	1/1(100.0)	0/0(0)	0/1(0)	0/0(0)	1/1(100.0)	0/0(0)	0/1(0)	0/0(0)
	SQ	chicken	2/5(40.0)	2/7(28.6)	1/5(20.0)	1/7(14.3)	5/5(100.0)	2/7(28.6)	5/5(100.0)	2/7(28.6)
	YC	chicken	1/11(9.1)	2/3(66.7)	0/11(0)	1/3(33.3)	7/11(63.6)	1/3(33.3)	3/11(27.3)	2/3(66.7)
		swine	1/1(100.0)	11/22(50.0)	0/1(0)	11/22(50.0)	1/1(100.0)	11/22(50.0)	1/1(100.0)	10/22(45.5)
	CZ	chicken	5/5(100.0)	2/4(50.0)	1/5(20.0)	2/4(50.0)	5/5(100.0)	3/4(75.0)	2/5(40.0)	2/4(50.0)
	NT	chicken	2/2(100.0)	1/3(33.3)	2/2(100.0)	0/3(0)	2/2(100.0)	3/3(100.0)	2/2(100.0)	2/3(66.7)
Total			16/44(36.4)	23/49(46.9)	4/44(9.1)	15/49(30.6)	38/44(86.4)	30/49(61.2)	14/44(31.8)	24/49(49.0)
			39/93 (41.9)		19/93 (20.4)		68/93 (73.1)		38/93 (40.9)	
Total			29/95(30.5)	38/90(42.2)	5/95(5.3)	22/90(24.4)	59/95(62.1)	62/90(68.9)	17/95(17.9)	50/90(55.6)
			67/185(36.2)		27/185(14.6)		121/185(65.4)		67/185(36.2)	

### Presence of Aminoglycoside Resistance Genes

Various aminoglycoside resistance genes were examined by PCR in all 185 *Campylobacter* isolates. The prevalence of the *aph(2")-If* gene was 57.6% (53/92) and 73.1% (68/93) in *Campylobacter* isolates in 2017 and 2018, respectively. Moreover, *aph(2")-If* gene was more prevalent in *C. coli* than in *C. jejuni* in 2017. Similar to the *aph(2")-If* gene described above, *aac(6')-Ie/aph(2")-Ia* gene was identified in 31.5% (63.4% in *C. coli* and 5.9% in *C. jejuni*, 2017) and 40.9% (49.0% in *C. coli* and 31.8% in *C. jejuni*, 2018), showing its common presence and higher prevalence in *C. coli*. Among all gentamicin-resistant strains, 44.8% (30/67) contained two of the resistant genes, and 14.9% (10/67) did not have any of these four tested genes. The prevalence of the *aadE-sat4-aphA-3* cluster in *Campylobacter* isolates was identified to be ≤10% in 2017, but it increased to about 20% in 2018 (Table 2).

### Transfer of Aminoglycoside Resistance Through Natural Transformation

The genomic DNAs of the two strains with high resistance to gentamicin (*C. jejuni* 165 and *C. coli* 254) were used as the donor DNA to transform the recipient strain (*C. jejuni* NCTC11168) by natural transformation under laboratory conditions. Compared with NCTC11168, the transformants NT-165 and NT-254 were obtained, showing 512- and 128-fold increases in the MICs of gentamicin, kanamycin, and tobramycin, respectively. The transformant NT-254 showed slightly decreased susceptibility to amikacin. However, the transformant NT-165 did not show any resistance to amikacin or streptomycin. The MICs of gentamicin, kanamycin, tobramycin, amikacin, and streptomycin in the recipient, donors, and transformants are revealed in Table 3.

**Table 3:** MICs of aminoglycosides in the transformants and donor strains.

Antimicrobial agent	MIC (μg/ml)				
	NCTC11168	165	NT-165	254	NT-254
Gentamicin	1	512	512	512	512
Kanamycin	8	1,024	1,024	1,024	1,024
Tobramycin	2	256	256	256	256
Amikacin	4	256	4	32	16
Streptomycin	4	256	4	4	4

*NT-165 and NT-254 are transformants of NCTC11168 with donor DNA from C. jejuni 165 and C. coli 254, respectively*.

**Figure 2:**
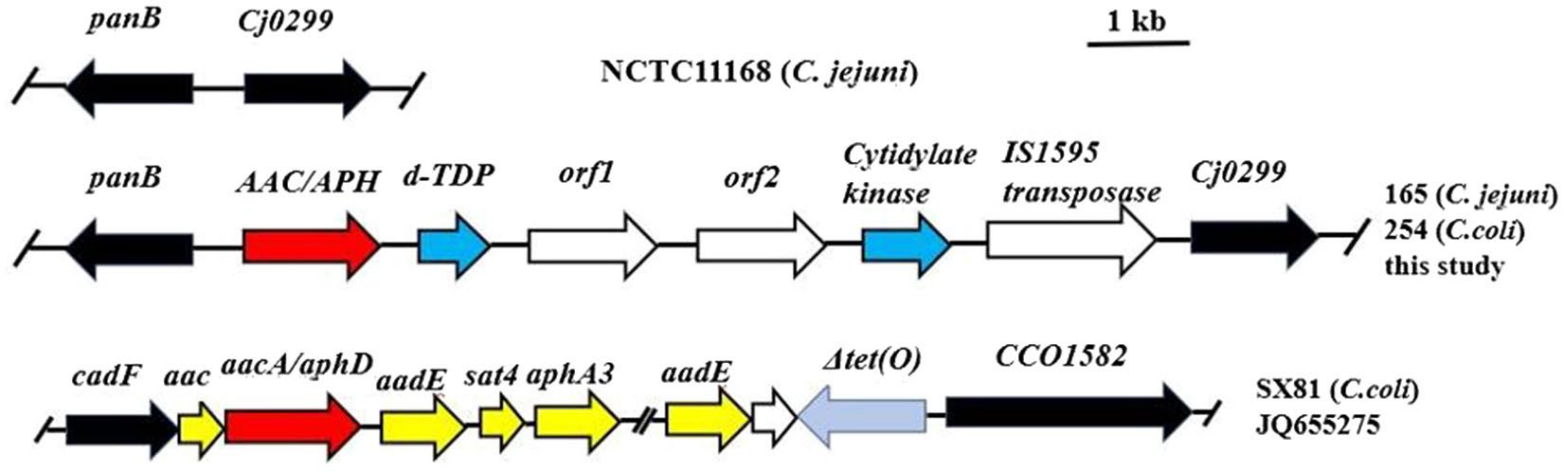
Genomic organization of the aminoglycoside resistance insertion segment in *C. jejuni* 165 and *C. coli* 254 in comparison with the multidrug resistance genomic island of *C. coli* SX81 and *C. jejuni* 11,168. Arrows indicate the positions and directions of transcription of the genes. The locations of primers *panB*-F and *cj0299*-R used to detect the insertion segment are indicated.

The two transformants were then investigated by whole-genome sequencing. Subsequently, the draft genomes of NT-165 and NT-254 were compared with NCTC11168, showing that the backbones of the transformants were NCTC11168. Moreover, a 5,200-bp segment was inserted between the *Campylobacter* highly conserved genes *Cj0299* and *panB*. The same insertion region was already identified in 2017 (Yao et al., 2017). The inserted segment contained six open reading frames (ORFs). It included genes encoding AAC/APH (a bifunctional enzyme), dTDP-fucosamine acetyltransferase, cytidylate kinase, IS1595 family transposase, and two hypothetical protein genes (Figure 2). Combined with the MICs results of the transformants above, it suggested that the six-gene cluster can transferred among *Campylobacter* strains naturally, and it conferred a high-level resistance to aminoglycoside antibiotics.

### Molecular Typing and Aminoglycoside Resistance of the Insertion-Fragment Positive *Campylobacter* Isolates

**Figure 3:**
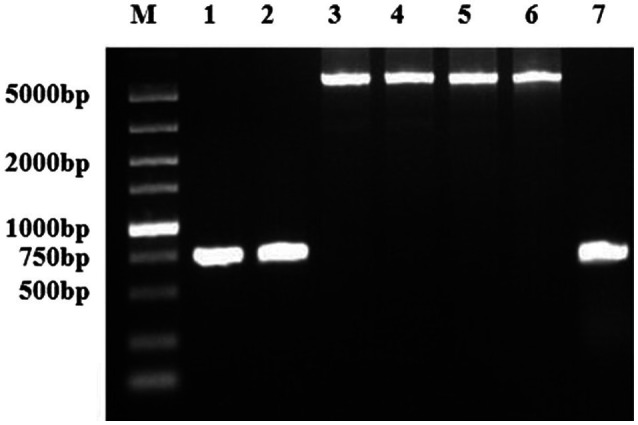
PCR detection of the region between *panB* and *Cj0299* in various strains. Lanes 1 and 2, PCR products from aminoglycoside-susceptible strains 28 and 31, respectively; Lanes 3 and 4, PCR products from the *C. jejuni* transformants of NT-165 and NT-254, respectively; Lanes 5 and 6, PCR products from aminoglycoside-resistant strains 165 and 254, respectively; and Lane 7, PCR products from *C. jejuni* NCTC11168.

Using the primers *cj0299*-F and *panB*-R, a 5.2-kb fragment was amplified in 9.7% (18/185) *Campylobacter* isolates. Some examined isolates, including aminoglycoside- resistant isolates and susceptible ones, did not yield this 5.2-kb fragment but showed a 750-bp fragment (Figure 3). These positive strains originated from five different locations in Jiangsu province (Table 4).

**Table 4:** MICs of aminoglycoside antibiotics and STs for insertion-fragment positive isolates.

Strain	Species	Year	Location	MIC (μg/ml)					ST	ST-clonal
				Gen	K	AK	TOB	S		
165	*C.jejuni*	2017	HA	512	1,024	256	256	256	new	–
254	*C. coli*	2017	HA	512	1,024	32	256	4	860	828
246	*C. coli*	2017	YC	256	512	64	64	2	10,062	–
1–1	*C.jejuni*	2018	CZ	128	1,024	128	64	128	9,627	828
1–2	*C.jejuni*	2018	CZ	128	1,024	64	64	128	9,627	828
2–1	*C.jejuni*	2018	CZ	128	1,024	128	64	128	9,627	828
2–2	*C.jejuni*	2018	CZ	128	1,024	128	64	64	9,627	828
52–1	*C.coli*	2018	NT	128	1,024	8	32	64	832	828
53–1	*C.jejuni*	2018	HA	64	1,024	16	64	32	829	828
55–1	*C.jejuni*	2018	YZ	128	512	64	64	128	10,062	–
58–3	*C. coli*	2018	YZ	256	1,024	128	64	32	10,062	–
58–4	*C. coli*	2018	YZ	256	1,024	128	64	64	10,062	–
58–6	*C. coli*	2018	YZ	256	1,024	64	64	64	10,062	–
58–8	*C.jejuni*	2018	YZ	128	512	64	64	64	10,062	–
59–2	*C. coli*	2018	YZ	512	1,024	64	64	64	1,666	828
61–4	*C.jejuni*	2018	YZ	128	1,024	64	64	128	10,062	–
62–2	*C.jejuni*	2018	YZ	256	1,024	128	64	64	10,062	–
62–4	*C. coli*	2018	YZ	256	1,024	128	64	64	10,062	–

Subsequently, the resistance to multiple aminoglycoside antimicrobials of the insertion-fragment positive strains was investigated. Most strains showed high MICs to gentamicin and kanamycin. About 72.2% (13/18) showed high MICs to all five tested aminoglycosides (Table 4).

To understand if the insertion-fragment positive isolates were genetically related, 18 isolates above were selected for MLST analysis. Seven STs were identified for these strains: a new ST (165 in location HA), ST860 and ST829 (254 and 53–1, respectively, in location HA), ST10062 (246 in location YC and 55–1, 58–3, 58–4, 58–6, 58–8, 61–4, 62–2, and 62–4 in location YZ), ST1666 (59–2 in location YZ), ST9627 (1–1, 1–2, 2–1, and 2–2 in location CZ), and ST832 (52–1 in location NT). Except for a new ST and ST10062 strains whose clonal complexes were not assigned, others belonged to clonal complex 828. ST10062 appeared in different years and different locations, although the number of ST10062 strains in 2017 was relatively small (Table 4).

## Discussion

Aminoglycosides use in veterinary medicine is associated with increased resistance to aminoglycosides and other antimicrobial classes in bacteria from animals. *C. jejuni* and *C. coli* isolates of clinical and animal origin display resistance to aminoglycoside streptomycin (van Duijkeren et al., 2019). In China, antimicrobial use records reveal that aminoglycoside agents, such as amikacin and neomycin, are commonly used to prevent bacterial diseases in food-producing animals (Wang et al., 2016). Although the level of resistance to gentamicin in *Campylobacter* is low in other countries, an increasing trend has been observed in recent years, and the gentamicin resistance rate of *Campylobacter* in China has become higher than those in other countries (Zhao et al., 2015; Yao et al., 2017; DANMAP, 2019). In the present study, the prevalence of gentamicin and kanamycin resistance in *Campylobacter* is ≥30.0%, consistent with previous reports in China (Qin et al., 2012; Ma et al., 2014; Yao et al., 2017). Previous studies have shown that gentamicin resistance is much more prevalent in *C. coli* than in *C. jejuni* strains (Wang et al., 2016; Yao et al., 2017). In the present study, a higher percentage of resistance to gentamicin and kanamycin was found in *C. coli* than in *C. jejuni* isolates. The resistance to gentamicin and kanamycin of *C. jejuni* and *C. coli* increased by more than 10.0% in 2018 compared with that in 2017 in Jiangsu province. The increasing trend suggested that we should pay attention to rational drug use and strengthen the monitoring of aminoglycoside drug resistance in China.

Resistance genes can be located on the plasmids, chromosome, transposons, or other mobile elements, increasing the aminoglycoside resistance and the co-resistance to other compounds (Ramirez et al., 2013). There is an apparent trend toward multidrug resistance, particularly among *C. coli* which harbored different antimicrobial resistance genes within the genome of a single isolate (European Food Safety Authority (EFSA), 2019). Four variants of aminoglycoside-resistant genes were analyzed by PCR. The bifunctional enzyme aac(6')-Ie/aph(2")-Ia confers resistance to almost all aminoglycosides except streptomycin (Zhao et al., 2015). In clinical *Enterococcus* isolates, it is the most important enzyme associated with high-level gentamicin resistance (Toth et al., 2013). A previous study has reported the recent emergence of *aph(2")-If* and has become the predominant gentamicin resistance determinant in *Campylobacter* in Shandong province in China (Yao et al., 2017). In the present study, the prevalence rates of the *aac(6')-Ie/aph(2")-Ia* gene in *Campylobacter* isolates were 31.5% and 40.9% in 2017 and 2018, respectively. A*ph(2")-If* gene was also more prevalent in *Campylobacter* herein than in another previous study in China. The reason for the increased prevalence of these two genes may be the different years and regions of the isolated strains. Further studies are needed to show if an annual increasing trend occurs in the presence of these two gentamicin resistance genes.

The gene cluster *aadE-sat4-aphA-3*, which confers resistance to streptomycin, streptothricin, and kanamycin, has been detected on a plasmid and in the chromosome in *Campylobacter* (Chen et al., 2013; Zhao et al., 2015). It was identified in a *C. coli* genomic island which harbors genes conferring resistance to multiple aminoglycoside antibiotics in China (Qin et al., 2012). The *aadE-sat4-aphA-3* gene cluster was observed in about ≤15.0% isolates in the present study. Among all cluster-positive strains, 13 isolates did not show any resistance to gentamicin or kanamycin. This result was not consistent with the previously reported results showing that all clusters carrying *C. coli* were resistant to gentamicin (Qin et al., 2012). But in another paper, the florfenicol-resistant gene *cfr(C)*-carrying *C. coli* isolates were susceptible to chloramphenicol and florfenicol (Liu et al., 2019). The gentamicin resistance gene *aacA4* has been reported in *C. jejuni* isolated from the water lines of a broiler-chicken house environment, and it is associated with class 1 integron (Lee et al., 2002). However, no *aacA4* gene was detected in the *Campylobacter* isolates examined in this study, consistent with a previous one (Yao et al., 2017). About 15.0% (10/67) gentamicin-resistant isolates contained none of the above four detected aminoglycoside-resistant genes which may harbor other unknown resistance mechanisms.

*Campylobacter* are well known for their ability to acquire exogenous DNA by natural transformation (Wang and Taylor, 1990). Some genes associated with high-level gentamicin resistance in *Campylobacter* have probably resulted from horizontal transfer from other microorganisms (Zhao et al., 2015). Herein, transformants with ≥128-fold increase in the MICs of gentamicin were obtained by natural transformation under laboratory conditions. This difference in aminoglycoside MIC values suggested that some genes of the genome were transferred from *C. jejuni* or *C. coli* to *C. jejuni* and intensively increased the aminoglycoside resistance in the recipient strain *C. jejuni*.

Genomic islands harboring aminoglycoside resistance genes and multidrug resistance have been previously detected between *cadF* and *CCO1582* on the chromosome in *C. coli*, and multiple aminoglycoside resistance genes have been found to be located between *Cj0299* and *panB* (Qin et al., 2012; Wang et al., 2014; Yao et al., 2017). Herein, we compared the draft genomes of two transformants with NCTC11168 and found a 5,200-bp segment inserted between the highly conserved *Campylobacter* genes *Cj0299* and *panB*. The presence of this segment was associated with elevated MIC values for aminoglycosides. In recent years, different multidrug resistance genomic islands (MDRGIs) conferring resistance to aminoglycosides, macrolides, and tetracyclines were characterized (Qin et al., 2012; Wang et al., 2014; Liu et al., 2019). Unlike MDRGIs in previous reports, the inserted segment in this study just contained six ORFs (Figure 2). *AAC/APH* which encoded a bifunctional enzyme was in it. Gene *aacA-aphD* was the only gene identified to encode a bifunctional aminoglycoside-modifying enzyme responsible for gentamicin and kanamycin resistance in *Campylobacter*. This insertion fragment could spread by horizontal gene transfer. In 18 insertion-fragment positive strains, about 72.0% (13/18) showed high MICs to all five tested aminoglycosides (Table 4). This result suggested that the insertion fragment was associated with aminoglycoside resistance, the prevalence of which could confer a fitness advantage under selection by continued aminoglycoside usage.

In a previous study, an aminoglycoside resistance island was reported to spread by both horizontal gene transfer and clonal expansion by PFGE and MLST analysis in Shandong province, China (Qin et al., 2012). To understand if the insertion-fragment positive *Campylobacter* isolates in the present study were genetically related or had any clonal expansion, 18 fragment positive isolates were selected for MLST analysis. Seven STs were identified for these strains. The 18 strains had no particular clonal expansion. Except for a new ST and ST10062 whose clonal complexes had not been assigned, all other strains belonged to clonal complex 828, consistent with a previous study (Qin et al., 2012). Interestingly, ST10062 appeared in different years and locations. More strains are needed to further determine whether this type of ST is a special clonal expansion for the dissemination of aminoglycoside resistance in China.

## Conclusion

This study provided the prevalence of gentamicin and kanamycin resistance and the associated aminoglycoside resistance genes in *Campylobacter* isolated from chicken and swine in Jiangsu province, China. A gene segment which could drastically increase aminoglycoside resistance by natural transformation was found. Owing to the use of aminoglycosides in poultry and swine production, *Campylobacter* in poultry and swine may be able to deal with the toxicity and selective pressure from these antibiotics. These findings offered insights into the prevalence and spread of the aminoglycoside resistance of *Campylobacter* in China, thereby highlighting the need for concerning and taking measures to reduce the dissemination of aminoglycoside resistance in *Campylobacter*.

## Data Availability Statement

The datasets presented in this study can be found in online repositories. The names of the repository/repositories and accession number(s) can be found at: MZ593442-MZ593447.

## Ethics Statement

This study was carried out in accordance with the principles of the Animal Welfare and Ethical Censor Committee of Jiangsu Institute of Poultry Science. No chickens and pigs were killed for the present study. When collecting cloacal swabs, well-trained farm workers hold the chickens. Fresh feces from pigs were collected without any manipulation of the pigs.

## Author Contributions

QZ, JZ, and JP performed the antibiotic susceptibility tests. XZ and QZ did the aminoglycoside genes detection. MT and XZ performed natural transformation and MLST. YZ and JL did the data analysis. XZ prepared the manuscript. YG supervised and assisted in the manuscript preparation. All authors contributed to the article and approved the submitted version.

## Funding

This work was supported by Yangzhou social development project (YZ2020061) and National Natural Science Foundation of China grant (31700005).

## Conflict of Interest

The authors declare that the research was conducted in the absence of any commercial or financial relationships that could be construed as a potential conflict of interest.

## Publisher’s Note

All claims expressed in this article are solely those of the authors and do not necessarily represent those of their affiliated organizations, or those of the publisher, the editors and the reviewers. Any product that may be evaluated in this article, or claim that may be made by its manufacturer, is not guaranteed or endorsed by the publisher.
